# Ultra-processed food exposure and health outcomes: Current evidence, controversies, and future perspectives

**DOI:** 10.17179/excli2026-9423

**Published:** 2026-07-09

**Authors:** Monica Dinu, Sofia Lotti, Farzaneh Asoudeh, Deb Junyi Zhang, Barbara Colombini, Sarah Gauci, Daniela Martini, Wolfgang Marx

**Affiliations:** 1 Department of Experimental and Clinical Medicine, University of Florence, 50134, Florence, Italy; 2 Deakin University, IMPACT, the Institute for Mental and Physical Health and Clinical Translation, Food & Mood Centre, School of Medicine, Barwon Health, Geelong, 3220, Australia; 3 Division of Human Nutrition, Department of Food, Environmental and Nutritional Sciences (DeFENS), University of Milan, 20133, Milan, Italy

**Keywords:** UPFs, Nova classification, cardiometabolic health, mental health, dietary guidelines, food processing

## Abstract

Ultra-processed food (UPF) exposure has increased markedly worldwide and is now a major contributor to total energy intake in many countries. Evidence from large prospective cohort studies suggests that higher UPF exposure is associated with adverse health outcomes, including overweight and obesity, type 2 diabetes, cardiovascular disease, selected cancers, depression, and increased all-cause mortality. Short-term experimental studies further indicate that diets rich in UPFs can promote excess energy intake and weight gain, supporting biological plausibility. Several mechanisms have been proposed, including poor nutrient quality, high glycemic load, altered food structure and satiety signaling, exposure to additives and contaminants, gut microbiome perturbations, and displacement of minimally processed foods. Nevertheless, the field remains debated. Major controversies concern the Nova classification system, substantial heterogeneity within the UPF category, challenges in reproducible classification, and the extent to which observed associations reflect processing itself rather than nutrient profile, dietary patterns, or broader food environments. These issues hinder causal inference and sustain ongoing debate over whether UPFs act as independent etiological factors or function as markers of unhealthy diets. Policy responses vary internationally, ranging from explicit inclusion of UPFs in dietary guidelines to predominantly nutrient-based or hybrid frameworks. This narrative review summarizes current evidence linking UPF exposure to health outcomes, critically examines methodological and conceptual limitations, and discusses implications for future research and public health policy.

See also the graphical abstract(Fig. 1).

## Introduction

Ultra-processed foods (UPFs) have rapidly become a dominant component of modern diets, raising concern about their impact on human health. These foods, ranging from sugary drinks and packaged snacks to plant-based meat alternatives and ready-to-eat meals, are increasingly displacing fresh, whole foods in many countries (Monteiro et al., 2025[113]). In the United States (US) and the United Kingdom (UK), more than half of the average diet now comes from UPFs, with some population groups, such as younger individuals or those with lower socioeconomic status, obtaining up to 80 % of their calories from these products (Martini et al., 2021[100]; Monteiro et al., 2025[113]).

These marked shifts in dietary patterns have stimulated growing research interest in the potential contribution of UPFs to rising rates of obesity and other adverse health outcomes worldwide. Recent large-scale systematic reviews, including umbrella reviews and meta-analyses, have underscored the public health relevance of UPFs, reporting consistent associations between high UPF intake and elevated risks of multiple adverse health outcomes (Lane et al., 2024[79]; Monteiro et al., 2025[113]). At the same time, the concept of “ultra-processed food” itself, including how it is defined within the Nova classification system and its relevance for nutritional research and policy, remains the subject of active scientific debate (Visioli et al., 2025[156]; Rezende et al., 2026[132]; Scrinis et al., 2025[139]; Hess et al., 2024[61]). Several countries have already incorporated food processing into national dietary guidelines (Koios et al., 2022[78]; Monteiro et al., 2025[113]), and concerns about highly processed foods have also influenced recent policy discussions in the US, including those informing the latest Dietary Guidelines for Americans (US Department of Agriculture, 2026[154]).

This narrative review provides a state-of-the-art overview of the association between UPF exposure and health outcomes, critically examines key controversies, including limitations of the Nova classification system, highlights differences in perspectives across countries, and outlines priorities for future research and public health action.

## The Nova Classification

UPFs are defined by the Nova classification system as industrially formulated products made from multiple industrial ingredients and processing steps, with little to no intact whole foods remaining (Monteiro et al., 2019[112]). Within the Nova framework, foods are classified into four groups according to the extent and purpose of processing (Table 1(Tab. 1)). What distinguishes UPFs is not food processing per se, but rather the extent, nature, and underlying purpose of processing (Sadler et al., 2021[136]). Monteiro and colleagues describe ultra-processing as a set of industrial practices designed to produce highly palatable, market-oriented products, which are typically energy-dense, rich in added sugars, unhealthy fats, and sodium, and low in fiber and micronutrients (Monteiro et al., 2019[112]). These products also commonly contain cosmetic additives designed to enhance sensory appeal and shelf life (Monteiro et al., 2025[113]). UPFs are generally ready to consume, require minimal preparation, and are engineered to facilitate rapid ingestion, a combination of characteristics that contributes to their increasing displacement of freshly prepared dishes and meals in contemporary diets (Monteiro et al., 2025[113]).

## Global Trends in UPF Exposure

Exposure to UPFs has increased markedly worldwide over recent decades, driven by the industrialization of food systems, globalization of supply chains, and changes in lifestyles and eating practices (Monteiro et al., 2025[113]). High-income Western countries, including the US, the UK, Canada, and Australia, were early adopters and currently exhibit the highest dietary shares of UPFs (Monteiro et al., 2025[113]; Marchese et al., 2022[98]). In these settings, UPFs are ubiquitous, inexpensive, and intensively marketed, and among certain subpopulations, particularly socioeconomically disadvantaged groups, they account for a large proportion of total energy intake (Dicken et al., 2024[36]). Particular concern has been raised regarding children and adolescents, among whom UPFs often constitute a substantial share of daily energy intake from an early age (Du et al., 2024[40]; Chen et al., 2025[24]; Conway et al., 2024[26]; United Nations Children's Fund, 2025[153]). Early-life exposure may have lasting implications, as diets rich in UPFs may shape taste preferences, reinforce liking for sweet, salty, and energy-dense foods, and influence long-term eating behaviors and food choices (Chamarthi et al., 2025[18]; The Lancet Child Adolescent Health, 2026[148]). In addition, children and adolescents are disproportionately exposed to intensive marketing of UPFs through television, digital platforms, product packaging, and school-related environments, which further normalizes frequent consumption of highly palatable, branded products (Ilieva et al., 2025[67]; Scrinis et al., 2025[139]).

In contrast, countries with strong traditions of home cooking and greater reliance on fresh or minimally processed foods, particularly in the Mediterranean region and parts of Asia and Latin America, have historically reported lower UPF exposure (Watanabe et al., 2024[162]). Within Europe, a clear north-south gradient is evident, with UPFs contributing approximately 10-25 % of total energy intake in countries such as Italy and Portugal, compared with around 40 % or more in the UK and Germany (Mertens et al., 2022[105]; Martini et al., 2021[100]; Monteiro et al., 2025[113]). Brazil represents an intermediate case, with UPFs contributing roughly 20-25 % of total energy intake, a proportion that is steadily increasing and raising concerns about the double burden of malnutrition (Monteiro et al., 2025[113]).

Rapid expansion of UPF markets is now occurring in many middle-income countries across Latin America, Asia, and Africa, where traditional dietary patterns are increasingly displaced by mass-produced foods such as sugar-sweetened beverages, instant noodles, and packaged snacks, driven by convenience and intensive marketing (Monteiro et al., 2025[113]). These products are often adapted to local tastes and promoted extensively by multinational food corporations (Baker et al., 2025[7]). Recent analyses, including those from the Lancet series, emphasize the role of profit-oriented industry practices in driving global increases in UPF sales, including pervasive advertising to children and adolescents and sustained opposition to public health regulation (Monteiro et al., 2025[113]; Scrinis et al., 2025[139]; Baker et al., 2025[7]). Consequently, UPF exposure is increasingly viewed not only as a nutritional concern, but also as an issue of corporate and policy influence within global food systems (Scrinis et al., 2025[139]; Baker et al., 2025[7]).

## Health Impacts of UPFs

Over the past decade, a large body of epidemiological research has consistently linked higher exposure to UPFs with increased risk of obesity, metabolic disorders, cardiovascular disease, cancer, mental health outcomes, and all-cause mortality (Lane et al., 2024[79]; Monteiro et al., 2025[113]). These associations have been reported across diverse populations and often persist after adjustment for overall diet quality and sociodemographic factors, suggesting that UPF exposure may be associated with health risk beyond conventional nutrient-based explanations. However, the strength and interpretation of these associations vary by outcome, exposure metric, and study design. Below, key findings are summarized by major health domains.

### Cardiometabolic health

Evidence linking UPF exposure to cardiometabolic outcomes is primarily derived from prospective observational studies. Across multiple cohorts, higher UPF exposure is consistently associated with greater weight gain over time and increased risk of overweight and obesity, with several studies reporting dose-response relationships across exposure categories (Machado et al., 2020[95]; De Amicis et al., 2022[30]; Moradi et al., 2023[115]; Vitale et al., 2024[157]; Lane et al., 2024[79]). Effect sizes, however, vary across studies, potentially reflecting differences in dietary assessment methods and the operationalization of the Nova classification. UPF exposure is most commonly expressed as the proportion of total energy intake derived from UPFs, although alternative metrics such as weight-based measures or servings have also been used.

Associations between UPF intake and type 2 diabetes have also been reported. In three large US cohorts, higher UPF intake was associated with increased diabetes incidence, independent of body mass index (BMI) and overall diet quality (Chen et al., 2023[23]). Similarly, in the EPIC cohort, each 10 % increase in energy intake from UPFs was associated with a 17 % higher risk of incident type 2 diabetes during long-term follow-up (Dicken et al., 2024[34]). Comparable findings have been observed in the UK Biobank, where diabetes risk increased per 10 percentage-point increment in UPF consumption assessed as a proportion of food weight (Levy et al., 2021[87]). At the evidence-synthesis level, the umbrella review by Lane et al. classified the association between UPF intake and type 2 diabetes as convincing, and a recent meta-analysis reported a 13 % higher diabetes risk for each 10 % increase in UPF consumption (Lane et al., 2024[79]; Souza et al., 2025[144]).

For cardiovascular outcomes, meta-analyses indicate a higher risk of hypertension, overall cardiovascular disease, and cerebrovascular disease among individuals with greater UPF consumption (Juul et al., 2021[73]; Chen et al., 2022[22]; Wang et al., 2022[159]; Yuan et al., 2023[169]; Qu et al., 2024[130]). Evidence is considered convincing for increased cardiovascular disease mortality, with up to a 50 % higher risk among individuals with the highest UPF exposure, and highly suggestive for increased all-cause mortality, with approximately 20 % higher risk (Lane et al., 2024[79]). A recent American Heart Association scientific advisory interpreted these associations largely as reflecting high intake of nutritionally poor UPFs that overlap with foods rich in unhealthy fats, added sugars, and sodium, while noting the need for further research to disentangle the potential roles of additives and food matrix alterations (Vadiveloo et al., 2025[155]). Further, while the advisory also reinforced existing cardiovascular guidance favoring minimally processed, nutrient-dense foods within overall dietary patterns, they acknowledged heterogeneity within the UPF category, noting that some affordable UPFs with more favorable nutritional profiles may be compatible with healthy diets (Vadiveloo et al., 2025[155]). Consistent with this view, emerging evidence suggests that associations differ by UPF subgroups, with stronger positive associations observed for sugar-sweetened or artificially sweetened beverages and processed meats than for other UPFs (Mendoza et al., 2024[103]), although the conceptual basis and methodological approaches underlying UPF subgroup analyses have also been challenged (Rezende et al., 2026[132]). An important evidence gap in the literature is that most studies focus on disease incidence, whereas data on prognosis, recovery, and outcomes among individuals with established conditions remain scarce, limiting inference for secondary prevention.

Clinical experimental evidence remains limited but is gradually expanding. Overall, differences between ultra-processed and minimally processed diets appear more consistent for energy intake and adiposity than for short-term cardiometabolic biomarkers. In the first randomized controlled trial (RCT), Hall et al. showed that participants consumed approximately 500 kcal/day more and gained about 0.9 kg over two weeks during the ad libitum ultra-processed diet phase compared with the minimally processed diet (Hall et al., 2019[56]). Despite clear differences in energy intake and body weight, short-term changes in most cardiometabolic biomarkers were modest. Although between-diet biomarker analyses were not reported, within-diet analyses indicated that triglycerides and high-density lipoprotein cholesterol (HDL) cholesterol decreased relative to baseline after both diets. During the minimally processed diet only, the inflammatory marker high-sensitivity C-reactive protein (hsCRP) and the homeostasis model assessment of insulin resistance (HOMA-IR), an index of insulin resistance derived from fasting glucose and insulin concentrations, decreased, while fasting glucose and insulin tended to decline. Notably, although the two diets were matched for energy and nutrient composition, fiber in the ultra-processed diet was incorporated into beverages rather than provided through whole foods, a difference that may have affected food texture and eating behavior.

More recently, the UPDATE trial extended this evidence to a free-living, community-based setting aligned with national dietary guidance (Dicken et al., 2025[35]). In this crossover RCT, adults with overweight or obesity followed ultra-processed and minimally processed diets consistent with the UK Eatwell Guide for eight weeks each. Greater reductions in body weight, BMI, fat mass, body fat percentage, and visceral adiposity were observed during the minimally processed diet, but these did not translate into broadly superior cardiometabolic improvements compared with the ultra-processed diet. Among cardiometabolic biomarkers, between-diet differences were observed for triglycerides and low-density lipoprotein (LDL) cholesterol: triglycerides decreased more during the minimally processed diet, whereas LDL cholesterol decreased less during the minimally processed diet than during the ultra-processed diet. Similar patterns have been reported by Hamano et al., who observed greater increases in liver enzymes (aspartate aminotransferase [AST], alanine aminotransferase [ALT], and gamma-glutamyl transpeptidase [γGTP]), markers of liver function and hepatocellular injury, during the UPF diet compared with the non-UPF diet (Hamano et al., 2024[57]). Between-diet differences were also observed in changes in total and HDL cholesterol, with greater increases during the UPF diet, whereas most other metabolic markers, including insulin resistance indices, did not differ between diets. Evidence from other community-based interventions is broadly consistent with these findings (Lopes et al., 2025[91]; Brandão et al., 2024[13]), and several ongoing RCTs are expected to further clarify effect sizes and relevance in real-world settings (Dinu et al., 2024[37]; Capra et al., 2024[15]; Lasschuijt et al., 2025[84]).

### Cancer

Observational evidence suggests that higher UPF consumption may be associated with increased cancer risk, although associations are generally site-specific and less consistent than those reported for cardiometabolic outcomes (DeVito and Sheean, 2026[33]). Early prospective evidence from the NutriNet-Santé cohort showed higher overall cancer incidence and breast cancer risk with increasing UPF exposure (Fiolet et al., 2018[46]). Subsequent analyses from the UK Biobank similarly identified positive associations between UPF intake and both overall cancer incidence and cancer mortality, with stronger signals for ovarian cancer incidence and for ovarian and breast cancer mortality (Chang et al., 2023[19]). In contrast, a more recent large pan-European analysis did not observe an association between UPF exposure and overall cancer mortality, highlighting substantial heterogeneity across populations, cancer sites, and exposure metrics (González-Gil et al., 2025[54]).

When examined specifically for breast cancer, findings are mixed. Several cohort studies reported higher breast cancer incidence and a trend toward increased breast cancer mortality with greater UPF intake, particularly among older women, alcohol consumers, and individuals with a family history of breast cancer (Chen et al., 2025[21]). However, evidence from meta-analyses indicates considerable variability. A recent meta-analysis reported a 25 % higher breast cancer risk associated with high consumption of fast foods and UPFs, although stronger associations were observed in case-control studies and in studies conducted in Latin America, where effect estimates ranged from approximately 65 % to 200 % higher risk. In contrast, associations did not reach statistical significance in prospective cohort studies or across menopausal strata (Karimi et al., 2025[75]). The meta-analysis also pooled studies assessing fast food consumption with those measuring UPF intake, which are related but conceptually distinct exposures and may contribute to heterogeneity in the pooled estimates.

For gastrointestinal cancers, a systematic review and meta-analysis of prospective cohort studies including more than 1.1 million participants found that the highest UPF exposure was associated with increased risk of colorectal cancer, colon cancer, and non-cardia gastric cancer (Meine et al., 2024[102]). In contrast, no associations were observed for hepatocellular, pancreatic, esophageal, gastric cardia, or rectal cancers (Meine et al., 2024[102]). Supporting a role in early colorectal carcinogenesis, a large prospective analysis within the Nurses' Health Study II reported a 45 % higher risk of early-onset conventional colorectal adenomas among participants with the highest UPF intake, independent of BMI and overall diet quality (Wang et al., 2026[158]). Indirect pathways may also contribute, as high UPF consumption is associated with obesity, a well-established risk factor for colorectal cancer (Tin et al., 2025[150]).

Evidence linking UPFs to liver cancer remains limited. Although higher UPF intake has been associated with increased long-term risk of metabolic dysfunction-associated steatotic liver disease, meta-analytic evidence does not support a clear association with primary liver cancer, including hepatocellular carcinoma or intrahepatic cholangiocarcinoma (Souza et al., 2025[143]). Additional site-specific evidence from the EPIC cohort indicates that higher UPF consumption is associated with increased risks of head and neck cancer and esophageal adenocarcinoma (Morales-Berstein et al., 2024[116]). Mediation analyses suggested that adiposity explained only a small proportion of these associations, pointing to the potential involvement of mechanisms beyond excess body fat.

Taken together, findings across cancer sites suggest that associations with UPF consumption are heterogeneous and appear to be site-specific rather than consistent across cancers. Some evidence also indicates stronger associations may be observed for specific UPF subgroups, such as sugar-sweetened beverages and ultra-processed meats, compared with the ultra-processed category as a whole (DeVito and Sheean, 2026[33]). Heterogeneity in exposure assessment, residual confounding, and the long latency of carcinogenesis continue to limit causal inference. These limitations underscore the need for mechanistic studies and long-term prospective research with improved characterization of UPF subtypes, processing-related exposures, and relevant intermediate pathways.

### Mental health, cognitive function, and neurodevelopment

Interest is growing in the potential role of overall diet quality, including the degree of food processing, in mental health and cognitive function. Multiple cohort and cross-sectional studies report positive associations between higher UPF intake and depressive outcomes (Adjibade et al., 2019[1]; Gómez-Donoso et al., 2020[53]; Lane et al., 2023[81]; Leal et al., 2023[85]; Samuthpongtorn et al., 2023[138]; Sun et al., 2023[146]; Werneck et al., 2024[165]), as well as anxiety and psychological distress (Amadieu et al., 2021[3]; Bonaccio et al., 2022[10]; Coletro et al., 2022[25]; Noll et al., 2022[124]; Lopes Cortes et al., 2021[90]; Gomes Gonçalves et al., 2023[52]; Bhave et al., 2024[9]). Recent meta-analytic evidence indicates that high adherence to an ultra-processed dietary pattern is associated with increased risk across multiple mental health outcomes (Lane et al., 2022[80]). However, depression is among the outcomes showing the most consistent associations across studies, with a pooled relative risk of 1.23 (95 % CI 1.08 to 1.39) (Monteiro et al., 2025[113]). Other meta-analytic evidence from an umbrella review also reported convincing evidence that higher UPF consumption was associated with prevalent anxiety outcomes (OR 1.48, 95 % CI 1.37 to 1.59) and combined common mental disorder outcomes (OR 1.53, 95 % CI 1.43 to 1.63) (Lane et al., 2024[79]). Most studies assess depressive outcomes using validated symptom scales or clinical diagnoses, whereas evidence for other psychiatric endpoints remains more heterogeneous. Emerging intervention evidence suggests that reductions in UPF intake may be associated with improvements in depressive symptoms among adults with major depressive disorder. In a secondary analysis of the SMILES trial, each 10 % reduction in the dietary share of UPFs was associated with an additional 2.5-point improvement in MADRS scores (Lane et al., 2023[82]).

Related work has also explored disordered eating patterns and addictive-like eating behaviors, which may both increase vulnerability to UPF overconsumption and reinforce habitual intake. A recent systematic review of nine cross-sectional studies reported that higher UPF intake was associated with bulimic-type eating disorders, binge eating, other non-restrictive eating disorders, and food addiction, whereas no consistent associations were observed with restrictive eating disorders or body dissatisfaction (Pereira et al., 2024[126]). The lack of longitudinal studies represents a major gap in understanding the directionality of these relationships. Conceptually, UPFs have been proposed to have addictive potential. Drawing on evidence from 281 studies across 36 countries, pooled prevalence estimates of food addiction have been reported at approximately 14 % in adults and 12 % in children, levels comparable to those observed for some legal substances (Gearhardt et al., 2023[49]). Certain UPFs may be particularly likely to elicit addictive-like responses due to specific macronutrient combinations, altered food matrices that accelerate carbohydrate and fat delivery, and the use of sensory additives that enhance palatability and reinforce consumption.

Emerging research has extended this literature to neurodevelopmental outcomes, with a focus on prenatal and early-life exposures. Higher maternal UPF intake has been associated with less favorable cognitive development in offspring (Zupo et al., 2024[170]; Mottis et al., 2025[117]), and some studies report positive associations between higher UPF consumption and attention-deficit/hyperactivity disorder (ADHD) in children and adolescents (Ferreira et al., 2024[45]; Yan et al., 2023[168]). However, this evidence base remains relatively sparse, relies almost exclusively on observational designs, and is therefore subject to residual confounding and limited causal inference.

Beyond psychiatric-related outcomes, several observational studies have examined cognitive function and neurodegenerative risk in relation to UPF intake. Higher consumption of UPFs has been associated with poorer cognitive performance and less favorable cognitive trajectories, although the evidence base remains limited and methodologically heterogeneous (Gomes Gonçalves et al., 2023[52]; Mottis et al., 2025[117]). Substantial variability exists in cognitive assessment tools and parameters, exposure metrics, and study populations, which are concentrated in a small number of geographic regions (Smith et al., 2025[140]). Associations with incident dementia, including Alzheimer's disease, have also been reported, suggesting a higher risk among individuals with greater UPF intake (Henney et al., 2024[60]; Weinstein et al., 2025[164]). Interpretation of these findings is complicated by the long preclinical phase of dementia and the potential for reverse causation, whereby early cognitive decline may influence dietary behaviors prior to clinical diagnosis.

Overall, the evidence linking UPF intake to mental and brain health is growing but remains predominantly observational. Residual confounding related to socioeconomic position and broader lifestyle factors, as well as bidirectional relationships between diet and mental health, remain key methodological challenges. Evidence is comparatively limited for psychiatric outcomes beyond depression and for endpoints other than binary measures of prevalence and incidence, such as analyses using continuous symptom measures, changes in symptom scores over time, clinical course, and long-term progression. Future research integrating longitudinal designs, repeated dietary and mental health assessments, and mechanistic approaches will be essential to clarify causal pathways and to better characterize the role of UPFs across the spectrum of mental and brain health.

### All-cause mortality and other health outcomes

In recent years, the range of health outcomes examined in relation to UPF exposure has expanded, often within the same large prospective cohorts. An updated dose-response meta-analysis of 18 cohort studies including more than 1.1 million participants reported a 15 % higher risk of all-cause mortality among individuals with the highest UPF intake, with a linear increase of approximately 10 % in mortality risk for each 10 % increment in UPF consumption (Liang et al., 2025[88]). Similarly, another dose-response meta-analysis estimated a 3 % increase in all-cause mortality per 10 % increase in dietary UPF share and suggested that a substantial proportion of premature deaths in countries with high UPF exposure may be attributable to these foods (Nilson et al., 2025[122]). Analyses from the UK Biobank further suggest that the association between UPF intake and mortality may be partly mediated by accelerated biological ageing, with differential effects observed across UPF subgroups, including beverages and products containing artificial sweeteners (Wang et al., 2025[161]).

Beyond mortality, evidence has also accumulated for gastrointestinal and renal outcomes. Meta-analytic data indicate that high UPF intake is associated with increased risk of inflammatory bowel disease, particularly Crohn's disease, with dose-response analyses supporting a positive linear association (Babaei et al., 2024[6]). A potential role of UPFs in irritable bowel syndrome has likewise been proposed, plausibly mediated by additive-related effects on gut microbiota composition, intestinal barrier function, and low-grade inflammation, although the current evidence base remains limited and largely observational (Dale et al., 2025[29]). For renal outcomes, systematic reviews and meta-analyses consistently report a higher risk of chronic kidney disease and declined renal function with greater UPF consumption, including dose-response relationships indicating increased risk with incremental UPF intake (Leonberg et al., 2025[86]; Hojjati Kermani et al., 2025[63]). These associations appear particularly relevant among vulnerable populations, such as older adults and individuals with pre-existing kidney disease (Kanbay et al., 2025[74]).

In the context of declining fertility rates in most industrialized countries, reproductive outcomes have begun to receive increasing attention in relation to UPF exposure (GBD 2021 Fertility and Forecasting Collaborators, 2024[48]). The current evidence remains limited and is derived predominantly from cross-sectional observational studies, which report associations between higher UPF intake and infertility, with BMI frequently identified as a potential mediating factor (Ceretti et al., 2024[17]; Evans et al., 2025[41]; Su et al., 2025[145]; Soltani et al., 2025[142]). Importantly, experimental evidence is now emerging. In a recently published randomized controlled 2-by-2 crossover feeding trial, consumption of an ultra-processed diet for 3 weeks resulted in adverse changes in body weight, lipid profiles, and endocrine markers, along with trends toward impaired sperm quality, despite equivalent energy intake across dietary conditions. Although preliminary, these findings provide experimental support for the possibility that UPFs may influence metabolic and reproductive health through mechanisms that extend beyond excess caloric intake alone (Preston et al., 2025[129]).

## Mechanistic Pathways Linking UPFs to Health Outcomes

**Figure 1:**
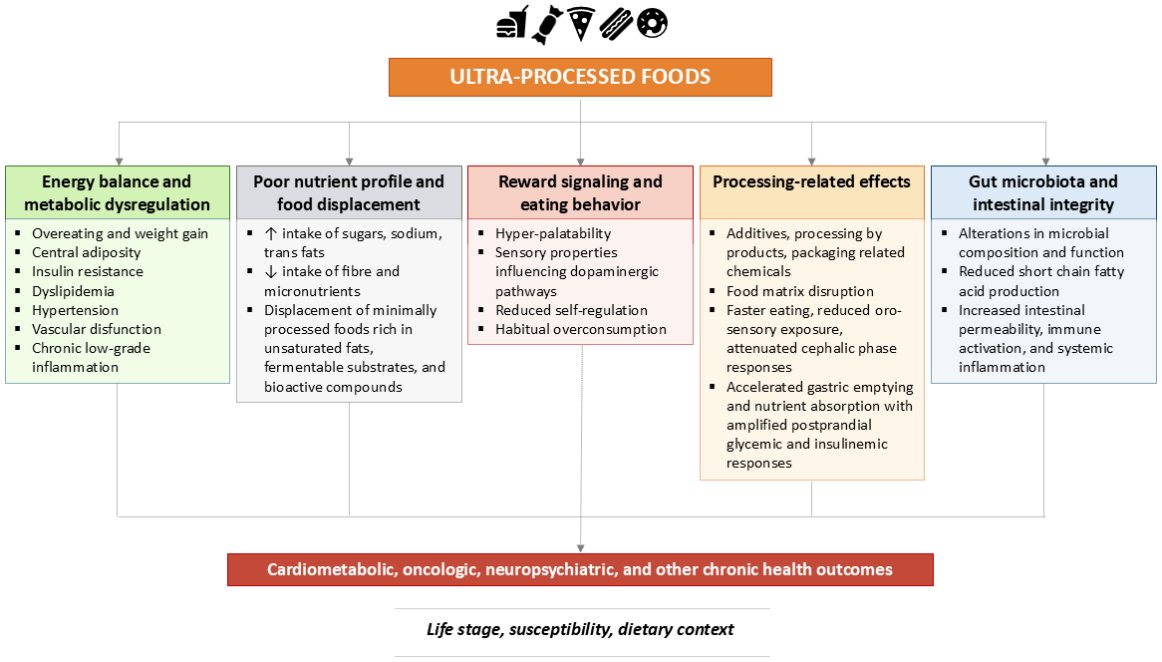
Graphical abstract: Proposed mechanistic pathways linking UPF consumption to adverse health outcomes. Multiple interacting mechanisms are depicted, including effects on energy balance, nutrient quality, eating behavior, food matrix structure, exposure to additives and processing-related compounds, and gut microbiota and intestinal function. These mechanisms are not mutually exclusive and may interact over time across behavioral, metabolic, immune, and neurobiological domains. Evidence strength varies across pathways, with the most consistent support for energy balance and nutrient-related mechanisms, while additive-, matrix-, and microbiota-related effects remain emerging. The relevance of specific pathways may further depend on life stage, individual susceptibility, and overall dietary context.

While these epidemiological and experimental findings provide converging evidence of associations between UPF exposure and adverse health outcomes, interpretation requires careful consideration of the biological pathways through which such effects might plausibly arise. Multiple mechanisms have been proposed, which are not mutually exclusive and are likely to interact over time (Figure 1(Fig. 1)). Together, these pathways may contribute to cardiometabolic, oncologic, neuropsychiatric, and other chronic conditions through a combination of relatively well-established processes and more speculative mechanisms for which direct human evidence remains limited (Robinson and Johnstone, 2024[133]).

A central and comparatively well-supported mechanism relates to energy balance and metabolic regulation. Diets high in UPFs are typically energy dense, rapidly consumed, and associated with higher total energy intake, weight gain, and central adiposity (Vitale et al., 2024[157]; Lane et al., 2024[79]; Monteiro et al., 2025[113]). Experimental evidence indicates that reformulation strategies may attenuate, but do not fully counteract, processing-related drivers of excess intake. In a controlled crossover study, a protein-enriched ultra-processed diet resulted in lower ad libitum energy intake and higher energy expenditure compared with a standard-protein ultra-processed diet, leading to a less positive energy balance; however, overeating persisted under both conditions (Hägele et al., 2025[55]). Changes in appetite-regulating hormones, including lower postprandial ghrelin and higher glucagon and peptide YY concentrations, suggest partial engagement of satiety pathways without normalization of intake (Hägele et al., 2025[55]). Over time, sustained excess energy intake and adiposity promote insulin resistance, dyslipidemia, vascular dysfunction, hypertension, and chronic low-grade inflammation (Tristan Asensi et al., 2023[151]), providing a shared mechanistic substrate for type 2 diabetes, cardiovascular disease, and several obesity-related cancers. Rapid postprandial glycemic excursions and repeated insulin stimulation associated with many UPFs may further exacerbate metabolic dysregulation, although direct causal evidence in humans remains limited (Vadiveloo et al., 2025[155]).

The typical nutrient profile of many UPFs represents another important and relatively well-characterized pathway. Diets high in UPFs are often rich in added sugars, sodium, and industrial trans-fatty acids, and low in fiber, potassium, and micronutrients, contributing to an overall unhealthy dietary pattern when intake is substantial (Vadiveloo et al., 2025[155]; Monteiro et al., 2025[113]). High consumption of UPFs may also generate displacement effects, whereby minimally processed, fibre- and micronutrient-rich foods are consumed less frequently, reducing intake of protective components such as unsaturated fatty acids, fermentable substrates, and bioactive compounds with anti-inflammatory and cardiometabolic benefits (Vadiveloo et al., 2025[155]). These mechanisms align with traditional dietary risk models and suggest that the health effects associated with UPF consumption may be largely mediated by nutrient composition. This interpretation partially contrasts with the original conceptual framing of the UPF hypothesis, which emphasized industrial processing itself as the primary driver of adverse health effects, largely independent of nutrient content (Monteiro, 2009[110]). In addition, other characteristics of UPFs, such as hyper-palatability and reward-enhancing properties, may influence dopaminergic signaling and eating behaviour, reinforcing habitual overconsumption (Hough et al., 2026[65]).

Another proposed pathway involves exposure to processing agents, additives, and by-products. UPFs commonly contain emulsifiers, stabilizers, colorants, flavor enhancers, and non-nutritive sweeteners, and may increase exposure to packaging-derived chemicals such as bisphenols and phthalates (Monteiro et al., 2025[113]; Muncke et al., 2025[118]). Concerns have been raised regarding potential “cocktail effects” resulting from chronic, low-dose exposure to multiple food additives and endocrine-disrupting chemicals that may act synergistically on metabolic, immune, and hormonal pathways (Payen de la Garanderie et al., 2025[125]; Recoules et al., 2025[131]). Experimental evidence supports the biological plausibility of such interactions. For example, recent in vitro screening of commonly consumed food additives and mixtures identified by the French NutriNet-Santé cohort found that while most individual additives were not genotoxic at the concentrations tested, certain mixtures exhibited genotoxic effects that could not be explained by the relative concentrations of the individual components, suggesting potential toxic synergies (Recoules et al., 2025[131]). However, evidence supporting these effects in humans remains limited, and direct causal links to clinical outcomes have yet to be firmly established (Robinson and Johnstone, 2024[133]).

Disruption of the food matrix through industrial processing has also emerged as a plausible mechanistic pathway (Monteiro et al., 2025[113]). The food matrix, defined as the physical and structural organization of nutrients within foods, plays a critical role in regulating digestion, nutrient release, satiety, and postprandial metabolic responses (Weaver and Givens, 2025[163]). Ultra-processing often breaks down natural cellular structures and recombines refined ingredients into soft, low-viscosity products with small particle size, facilitating rapid eating and reducing oro-sensory exposure. Faster eating rates and shortened oro-sensory stimulation may attenuate cephalic-phase responses and delay satiety signaling, contributing to passive overconsumption. In addition, altered physical structure can accelerate gastric emptying and nutrient absorption, amplifying postprandial glycemic and insulinemic responses (Mackie, 2024[96]).

Alterations in gut microbiota composition and function represent another potential pathway linking UPF intake to adverse health outcomes (Whelan et al., 2024[166]). Experimental evidence, largely derived from animal models, indicates that specific additives frequently present in UPFs may impair intestinal homeostasis. Emulsifiers such as carboxymethylcellulose and polysorbate-80 have been shown to alter gut microbial composition, disrupt intestinal barrier integrity, and promote low-grade inflammation (Delaroque et al., 2025[32]). In parallel, the reduced availability of fermentable substrates typical of UPF-rich diets may limit microbial production of short-chain fatty acids, which are essential for epithelial integrity, immune regulation, and metabolic homeostasis (Rondinella et al., 2025[135]). These changes may increase intestinal permeability and systemic exposure to bacterial components, contributing to chronic inflammation implicated in cardiometabolic disease, cancer progression, and neuropsychiatric outcomes (Tristan Asensi et al., 2023[151]; Maki et al., 2024[97]; Whelan et al., 2024[166]).

In humans, causal inference regarding microbiota-mediated mechanisms is constrained by substantial inter-individual variability and methodological challenges, including the predominance of compositional over functional analyses and the difficulty of distinguishing processing effects from overall dietary patterns. Nevertheless, intervention studies provide emerging, though still modest, support for microbiota-related pathways. In a RCT among individuals with obesity, restriction of UPFs to 5 % of total intake within an energy-restricted diet over six months was associated with greater increases in health-associated microbial taxa, including members of the Ruminococcaceae family and the *Faecalibacterium* genus, compared with a standard energy-restricted diet (de Oliveira et al., 2025[31]). Similarly, a trial comparing food-based and supplement-based very low-energy diets, which were broadly characterized as low- versus high-UPF, respectively, reported greater increases in microbial alpha diversity and more favorable compositional changes in the food-based group, despite similar weight loss and comparable nutrient intakes (Lane et al., 2025[83]). Together, these findings suggest that processing level and matrix integrity may influence gut microbiome responses, independent of energy intake and macronutrient composition.

Collectively, these interconnected pathways may act across behavioral, metabolic, immune, and psychological domains and are relevant to a broad spectrum of chronic diseases. At the same time, mechanistic evidence remains strongest for weight-related outcomes, along with energy balance and nutrient-related aspects of UPF-rich diets, whereas evidence for additive- and matrix-related mechanisms is still emerging. Importantly, the Nova classification is defined by the extent, purpose, and nature of processing rather than nutrient composition, underscoring the complexity of attributing effects to processing per se. This highlights the need to avoid both oversimplification and undue demonization of processing, while maintaining a focus on overall diet quality as a central determinant of health (Dinu et al., 2026[39]). The relevance of specific mechanisms is also likely to vary by outcome, life stage, and underlying susceptibility, which may partly explain heterogeneity across disease endpoints.

## Controversies and Debates: Ultra-Processed or just Unhealthy?

Key methodological limitations in UPF research, their implications, and proposed methodological advances are summarized in Table 2(Tab. 2). The main controversies do not concern the existence of associations with adverse health outcomes, but rather how these associations should be interpreted in relation to causality, classification, and policy translation (Gibney et al., 2017[51]; Jones, 2019[71]; Petrus et al., 2021[127]; Braesco et al., 2022[12]; Gibney, 2023[50]; Bradbury and Mackay, 2024[11]; Janssen, 2024[68]; Trumbo et al., 2024[152]; Louie, 2025[92]; Visioli et al., 2025[156]; The Lancet Gastroenterology Hepatology, 2025[149]). Central to this debate is whether reported health effects reflect properties of food processing or whether UPFs primarily act as proxies for unfavorable nutrient profiles and broader dietary patterns. This section synthesizes the principal areas of contention and discusses their implications for causal inference and policy relevance.

### Definition and heterogeneity of UPFs

A major critique is that the Nova system aggregates a highly heterogeneous range of foods into a single “ultra-processed” category, which may be overly broad and conceptually imprecise (Petrus et al., 2021[127]; Louie, 2025[92]). This concern relates primarily to the qualitative and descriptive nature of the Nova definition, which relies on criteria for the extent and purpose of industrial processing rather than quantitative thresholds. Consequently, the classification may introduce subjectivity and potential misclassification, particularly when detailed information on ingredients, formulation, brand names, or processing methods is lacking in dietary datasets (Monteiro et al., 2025[113]). This limitation is especially relevant for mixed dishes and multi-ingredient products and may contribute to between-study heterogeneity (Jung et al., 2025[72]). At the same time, more recent evaluations have reported acceptable construct validity and strong inter-coder agreement for the Nova classification system. Empirical assessments indicate that the system's definitions and examples allow classification of more than 70 % of food items reported in food frequency questionnaires from US cohorts and over 90 % of food items recorded in 24-hour dietary recalls from participants in a national Brazilian dietary survey (Khandpur et al., 2021[77]; Sneed et al., 2023[141]; Louzada et al., 2023[93]). In parallel, recent initiatives, including the development of best practice guidelines, have focused on improving the efficiency and transparency of Nova food group categorization, with the aim of enhancing the accuracy of effect estimates (Martinez-Steele et al., 2023[99]).

In addition, UPFs vary widely in composition, nutrient profiles, and food matrices, and treating them as a homogeneous exposure may obscure meaningful differences in underlying biological mechanisms (Wang et al., 2024[160]; Messina et al., 2023[106]; Visioli et al., 2025[156]). For example, sugar-sweetened beverages and processed meats are clearly ultra-processed and consistently associated with obesity, diabetes, and cardiovascular disease. In contrast, other UPFs, including certain plant-based or mixed products such as probiotic yogurts that may contain additives or added micronutrients, have shown neutral or even beneficial associations in some observational studies (Cordova et al., 2023[27]; Louie, 2025[92]). These contrasts suggest that UPFs with different nutrient compositions and ingredient origins may exert health effects through distinct pathways, and that pooling such products may conflate the effects of processing with those of nutritional quality (Louie, 2025[92]; Visioli et al., 2025[156]). In this context, others have argued that the primary relevance of the UPF construct lies in its role as a marker of dietary patterns characterised by industrial formulations, high energy density, and displacement of minimally processed foods, making comparisons between ultra-processed products themselves less informative than comparisons between UPF and minimally processed alternatives within similar food categories (Monteiro et al., 2026[114]). Indeed, to better isolate potential effects of ultra-processing, appropriate counterfactual comparisons would ideally contrast the same food type across different processing levels, for example, a fruit-flavored yoghurt drink versus plain yoghurt with fresh fruit (Rezende et al., 2026[132]).

More broadly, critics argue that research and policy should focus primarily on specific nutrients or ingredients of concern, such as added sugars, trans fats, and sodium, as well as on overall dietary patterns, rather than on the less clearly defined concept of ultra-processing. Proponents of the Nova framework counter that it should be viewed as a complementary analytical tool rather than a replacement for established nutrient- and pattern-based approaches. From this perspective, foods, nutrients, additives, and food matrices are all relevant determinants of health, and the contribution of UPFs reflects not only their nutrient profiles but also shared characteristics related to industrial formulation, altered food matrices, sensory engineering, and marketing practices that promote overconsumption (Monteiro et al., 2025[113]; Rezende et al., 2026[132]; Monteiro et al., 2026[114]). This debate remains active, and a notable exchange of viewpoints published in the American Journal of Clinical Nutrition highlights the persistent divide among nutrition scientists regarding the scope, limitations, and utility of the Nova classification (Astrup and Monteiro, 2022[5]; Monteiro and Astrup, 2022[111]).

### Methodological limitations in research

Despite consistent epidemiological associations between UPF exposure and adverse health outcomes, causal inference remains limited, and further mechanistic studies and RCTs are needed to clarify whether and how UPFs directly contribute to disease risk (Forde, 2023[47]; Visioli et al., 2025[156]). The current evidence base is dominated by observational studies, which are inherently vulnerable to residual confounding, as high UPF intake often co-occurs with other unhealthy behaviors and socioeconomic disadvantage. Although most studies adjust for a wide range of covariates, residual and unmeasured confounding cannot be excluded and may partially account for the observed associations. In this context, recent analyses suggest that unmeasured confounding of sufficient magnitude to account for associations between UPF intake and weight gain in longitudinal cohort studies is at least plausible, underscoring the need for more robust causal study designs (Robinson and Jones, 2024[134]).

Dietary assessment represents an additional and important source of uncertainty. Although some questionnaires have been specifically developed to assess the consumption of foods according to their level of processing (Dinu et al., 2021[38]; Neri et al., 2023[121]), commonly used tools such as food frequency questionnaires and 24-hour dietary recalls were not originally designed to capture the degree or purpose of food processing. Many also predate the development of the Nova classification, increasing the risk of exposure misclassification (Jung et al., 2025[72]). This raises some methodological questions. On one hand, misclassification has been suggested to be largely non-differential with respect to outcomes and would therefore be expected to bias associations toward the null. On the other hand, measurement error may disproportionately affect analyses of specific UPF subgroups compared with analyses of overall ultra-processed dietary patterns, as attempts to distinguish finer product categories using imperfect instruments may amplify instability and misclassification (Rezende et al., 2026[132]). Consistent with this interpretation, studies using dietary assessment tools specifically designed to capture food processing according to the Nova framework have generally reported stronger associations between UPF consumption and health outcomes (Werneck et al., 2024[165]).

Methodological innovations are beginning to address some of these limitations. Machine learning and artificial intelligence approaches are increasingly applied to automate and standardize Nova coding across large datasets, including efforts to leverage Food Composition Tables to incorporate more detailed product information, with the potential to improve reproducibility and reduce inter-researcher variability (Menichetti et al., 2023[104]; Campbell et al., 2026[14]; Cattem et al., 2026[16]). In addition, linking dietary intake data with food purchase and retail transaction datasets offers opportunities to quantify UPF exposure more objectively over time and across populations. Related approaches have already been implemented in large prospective cohorts such as the French NutriNet-Santé, where repeated 24-hour dietary records capturing branded food products are dynamically linked to multiple composition databases and laboratory assays to estimate individual-level exposure to food additives and other processing-related components (Chazelas et al., 2021[20]). However, such approaches may incompletely capture foods consumed outside the home or in informal food environments and do not fully resolve broader conceptual challenges related to heterogeneity within the ultra-processed category (Campbell et al., 2026[14]).

Selective reporting and publication bias may further bias the body of published evidence, as studies identifying harmful associations are more likely to attract attention (Louie, 2025[92]). Another important limitation is the scarcity of long-term intervention trials explicitly targeting UPF and clinical endpoints, mainly due to ethical, logistical, and financial constraints. Consequently, critics argue that the current evidence does not definitively establish UPFs as independent causal agents and that public health strategies focusing on established nutritional targets such as added sugars, sodium, and unhealthy fats may capture much of the observed risk without requiring a distinct ultra-processing framework (Astrup and Monteiro, 2022[4]; Braesco et al., 2022[12]; Visioli et al., 2025[156]). Finally, although several plausible biological mechanisms have been proposed, direct evidence linking specific UPF components to particular disease outcomes remains limited. Ongoing research examining additives such as emulsifiers and non-nutritive sweeteners, as well as processing-related byproducts such as advanced glycation end-products, and packaging-related chemicals aims to clarify their individual and combined contributions (Muncke et al., 2025[118]; Hasenböhler et al., 2026[58]; Salame et al., 2024[137]).

### Socioeconomic and cultural context

Concerns have also been raised about the practical and social implications of stigmatizing UPF consumption. As discussed above, UPFs are typically inexpensive, convenient, and shelf-stable, characteristics that make them especially relevant for lower-income groups and for individuals facing time, financial, or resource constraints (Coyle et al., 2022[28]; Baker et al., 2025[7]). These consumption patterns are linked with persistent gender inequalities, as women continue to bear a disproportionate share of responsibility for food provisioning and meal preparation in many settings, even when engaged in paid work (Njuki et al., 2023[123]). Time scarcity, caregiving demands, and unequal access to resources may therefore increase household reliance on ready-to-eat products, particularly in families with young children or dependent members. In this context, public health recommendations that broadly discourage all UPFs risk being unrealistic and potentially counterproductive if not accompanied by policies that improve access to affordable, nutritious alternatives (Forde, 2023[47]; Scrinis et al., 2025[139]). In food-insecure or highly urbanized environments, UPFs may partially compensate for limited food availability by providing low-cost calories that require minimal preparation. Discouraging their consumption without parallel structural changes to food systems may inadvertently increase the risk of inadequate dietary intake (Scrinis et al., 2025[139]).

Importantly, not all forms of food processing have been detrimental to public health. Within the Nova framework, fortification strategies aimed at restoring nutrients lost during processing, such as enrichment of wheat or corn flour with iron and folic acid, have been widely used in processed staple foods to prevent micronutrient deficiencies. Several successful public health interventions have relied on processed or fortified foods, including ultra-processed fortified cereals, to prevent or reduce micronutrient deficiencies at the population level (Hoffman et al., 2020[62]; Hossain et al., 2025[64]). While the presence of fortification alone does not determine whether a food is classified as ultra-processed within the Nova framework, concerns have been raised that overly restrictive interpretations of UPF reduction could inadvertently discourage the use of fortified or nutritionally enhanced products that play an important role in food security, particularly for women of reproductive age, children, and other nutritionally vulnerable groups (Forde, 2023[47]).

There is also a cultural dimension to consider. UPFs have become embedded in many contemporary food practices and culinary traditions, such as instant noodles in parts of Asia or packaged breads in Western diets. Strategies aimed at reducing UPF exposure must therefore account for cultural acceptability and feasibility and are more likely to be effective if they promote gradual, context-sensitive transitions rather than abrupt dietary shifts. Overall, policies targeting UPFs should be carefully designed to avoid stigmatizing lower-income groups and to ensure that healthier dietary patterns are both accessible and culturally appropriate (Popkin et al., 2021[128]; Scrinis et al., 2025[139]).

In light of these debates, several experts have argued for refining the UPF framework rather than abandoning it altogether (Sadler et al., 2021[136]; Ahrné et al., 2025[2]; Medin et al., 2025[101]; Bernstein et al., 2026[8]). Monteiro and colleagues have likewise acknowledged key limitations of the framework. In the recent Lancet series, they recognized the need for additional RCTs and mechanistic studies, as well as the heterogeneity within the UPF category and the fact that not all UPFs have equivalent nutritional value (Monteiro et al., 2025[113]). At the same time, they and others have argued that the current body of evidence is sufficiently robust to justify population-level actions to reduce UPF exposure and that placing emphasis on unresolved scientific uncertainties may delay policies with the potential to mitigate obesity and other chronic diseases (Baker et al., 2025[7]; Scrinis et al., 2025[139]).

From a policy perspective, this debate implies different strategic emphases. If processing-related characteristics exert effects independent of nutrient profile, food-based and processing-oriented recommendations may be warranted. Conversely, if UPFs primarily function as markers of poor nutrient quality and unhealthy dietary patterns, nutrient-focused or hybrid approaches may capture much of the relevant risk. Balancing timely action based on consistent but imperfect evidence with the need for stronger causal resolution therefore remains central to ongoing discussions surrounding UPFs.

## UPFs in Dietary Guidelines

National and international approaches to UPFs remain highly heterogeneous, reflecting persistent scientific debate regarding the conceptual clarity and policy usefulness of the Nova framework (FAO, 2021[43]; Scrinis et al., 2025[139]; Liuim et al., 2026[89]). An analysis of 106 national dietary guidelines found that approximately 45 % included some reference to food processing, most commonly through recommendations to limit specific products such as sugar-sweetened beverages, sweets, fast foods, and processed meats (Koios et al., 2022[78]). By contrast, only about 5 % issued explicit positive recommendations to prioritize fresh or minimally processed foods, typically framed in broad terms such as “choose fresh foods” or “avoid packaged foods” (Koios et al., 2022[78]).

Brazil was the first country to integrate food processing into its national dietary guidance. The 2014 Brazilian Dietary Guidelines explicitly recommend limiting UPFs while prioritizing fresh and minimally processed foods and home-prepared meals, marking a shift from nutrient-based to food- and processing-based guidance (Ministry of Health of Brazil, 2015[109]). Similar principles were subsequently adopted in several Latin American countries, including Ecuador, Chile, El Salvador, Peru, and Uruguay, influenced by the Nova classification (FAO, 2021[43]). These guidelines emphasize not only foods to limit, but also cooking practices, food culture, and the social dimensions of eating. Evaluations of related policy initiatives in the region, particularly Chile's integrated food environment regulations combining front-of-pack warning labels, marketing restrictions, and school food policies, suggest that such approaches can modify the food environment and consumer behaviour (Scrinis et al., 2025[139]). Documented effects include reformulation of packaged foods to reduce regulated nutrients, substantial reductions in marketing of unhealthy products to children, decreased availability of such products in schools, and measurable declines in purchased calories, sugars, saturated fat, and sodium. Nevertheless, longer-term evidence linking these policies to sustained changes in dietary patterns and health outcomes remains an active area of research.

In Europe, Belgium and Israel have adopted the most explicit policy positions, recommending minimizing UPF consumption and prioritizing unprocessed or minimally processed foods (Superior Health Council, 2025[147]; Ministry of Health, 2020[108]). In contrast, most European countries continue to rely predominantly on nutrient-focused dietary guidance. In North America, dietary guidance has historically been nutrient-centric, but recent documents suggest a gradual shift toward recognizing food processing as a dimension of diet quality. The 2025-2030 Dietary Guidelines for Americans emphasize diets based on “real foods” and recommend substantial reductions in highly processed products rich in refined carbohydrates, added sugars, sodium, unhealthy fats, and chemical additives (US Department of Agriculture, 2026[154]). Although Nova terminology is not used, these recommendations show conceptual alignment with concerns about ultra-processing. Similarly, Canada's Food Guide advises limiting highly processed foods and consuming them less often and in smaller amounts, conveying a message broadly consistent with the UPF concept without explicitly adopting the term (Health Canada, 2019[59]).

In Asia and the Pacific region, Malaysia (NCCFN, 2021[120]), New Zealand (Ministry of Health, 2020[107]), and India (ICMR-NIN Expert Committee, 2024[66]) explicitly reference food processing or the Nova classification in their dietary guidance, whereas many other countries do not. Chinese dietary guidelines emphasize limiting processed and high-salt foods and encourage fresh and home-prepared meals, primarily from a nutritional and culinary perspective rather than through a formal processing-based framework (FAO, 2022[42]). Australia has adopted a more cautious approach, remaining largely food-group and nutrient-based, with no explicit reference to UPFs or the Nova classification (National Health and Medical Research Council, 2013[119]). However, the Australian guidelines are currently under review, and UPFs have been identified as an emerging area of interest within the evidence evaluation process, with targeted calls for systematic reviews examining the role of UPF consumption in health outcomes. Nevertheless, UPFs are increasingly discussed in research and policy debates across the region, even where this has not yet translated into explicit processing-based recommendations (Machado et al., 2025[94]).

In Africa, UPF exposure has historically been lower than in high-income regions, reflecting greater reliance on traditional diets based on minimally processed staples. However, rapid urbanization, globalization of food markets, and the expansion of multinational food corporations are accelerating UPF penetration, particularly in urban areas (FAO, 2024[44]). This trend raises concern about a growing double burden of malnutrition, in which persistent undernutrition and micronutrient deficiencies coexist with rising obesity and cardiometabolic disease. Most African dietary guidelines remain primarily nutrient- and food-group-based and do not explicitly reference UPFs or the Nova classification, although many discourage consumption of sugar-sweetened beverages, highly refined foods, and packaged snacks, thereby indirectly targeting ultra-processed products (FAO, 2021[43]). Notable exceptions include Zambia, Ethiopia, Gabon, and South Africa, where more explicit references to food processing or the Nova framework are present (FAO, 2021[43]). In these contexts, policy approaches to UPFs must be carefully balanced against considerations of food security, affordability, and micronutrient adequacy.

At the global level, the WHO and the FAO have increasingly acknowledged the role of food processing in shaping dietary patterns. The Nova classification has been used in several WHO reports to describe global trends in food exposure, and WHO has recently convened a Guideline Development Group to develop specific recommendations on UPF exposure, formally recognizing UPFs as an issue of international public health relevance (WHO, 2025[167]). UNICEF has similarly expressed concern regarding UPF exposure in children and has supported regulatory measures such as restrictions on marketing to young populations (United Nations Children's Fund, 2025[153]). Together, these developments suggest that more explicit and potentially more harmonized guidance on UPFs may emerge at the global policy level in the coming years.

## Future Perspectives

Looking ahead, both research and policy would benefit from moving beyond documenting associations towards a more precise identification of the specific attributes of UPFs that are most strongly linked to health risk. Rather than treating UPFs as a homogeneous exposure, increasing attention is being directed to the relative contributions of disrupted food structure, rapid digestibility, additive exposure, altered satiety signaling, microbiota perturbation, and packaging-derived contaminants. A gradual shift from predominantly descriptive epidemiology toward more mechanistic and intervention-based research could help refine current interpretations and support the development of more targeted and biologically grounded prevention strategies.

In this context, emerging study designs that combine controlled feeding interventions with omics-based approaches may offer valuable insights into early metabolic, inflammatory, endocrine, and microbiome-related responses to UPF exposure. Short- and medium-term interventions that replace UPFs with minimally processed foods under real-life conditions could also provide informative evidence on the potential reversibility of metabolic and inflammatory alterations. Such approaches are generally more feasible than long-term trials with hard clinical endpoints, while still strengthening causal inference.

At the conceptual level, the Nova framework may increasingly serve not only as a classificatory tool but also as a hypothesis-generating model to investigate the biological and technological determinants underlying the observed health effects of UPFs. Integrating dimensions such as processing intensity, food matrix integrity, additive burden, and nutritional quality could allow for a more refined stratification of UPFs based on their biological plausibility of harm, rather than treating them as a single, uniform category. This perspective may support a gradual shift in policy emphasis, from nutrient-based reformulation alone toward greater consideration of technological practices, including the use of specific additives, aggressive sensory engineering, and high energy density formulations that may be particularly relevant to health risks.

Finally, UPFs can be interpreted as indicators of broader food system dynamics rather than solely the result of individual dietary choices. Their widespread consumption reflects structural drivers such as economic incentives favoring shelf-stable products, time constraints in daily life, and the gradual erosion of cooking skills and food culture. From this perspective, effective responses to UPF exposure are likely to involve systemic approaches that complement individual-level guidance, including reshaping food environments, strengthening regulations on marketing, particularly to children, and redefining convenience in ways that better support minimally processed foods.

Emerging intervention studies provide preliminary support for this approach. School-based programs combining nutrition education with behavioral or experiential components, such as gardening and cooking activities, have been associated with reduced UPF intake and increased consumption of minimally processed foods among children and adolescents in different settings, including India and the US (Jeans et al., 2023[70]; Kaur et al., 2026[76]). Similarly, a multicomponent intervention delivered through primary health care services in Brazil resulted in significant reductions in UPF consumption among children with obesity over nine months (Jardim et al., 2026[69]). Addressing UPF exposure requires coordinated action on food environments and social contexts, alongside efforts to ensure that minimally processed foods are accessible, culturally acceptable, and compatible with contemporary lifestyles.

## Conclusions

**Figure 2:**
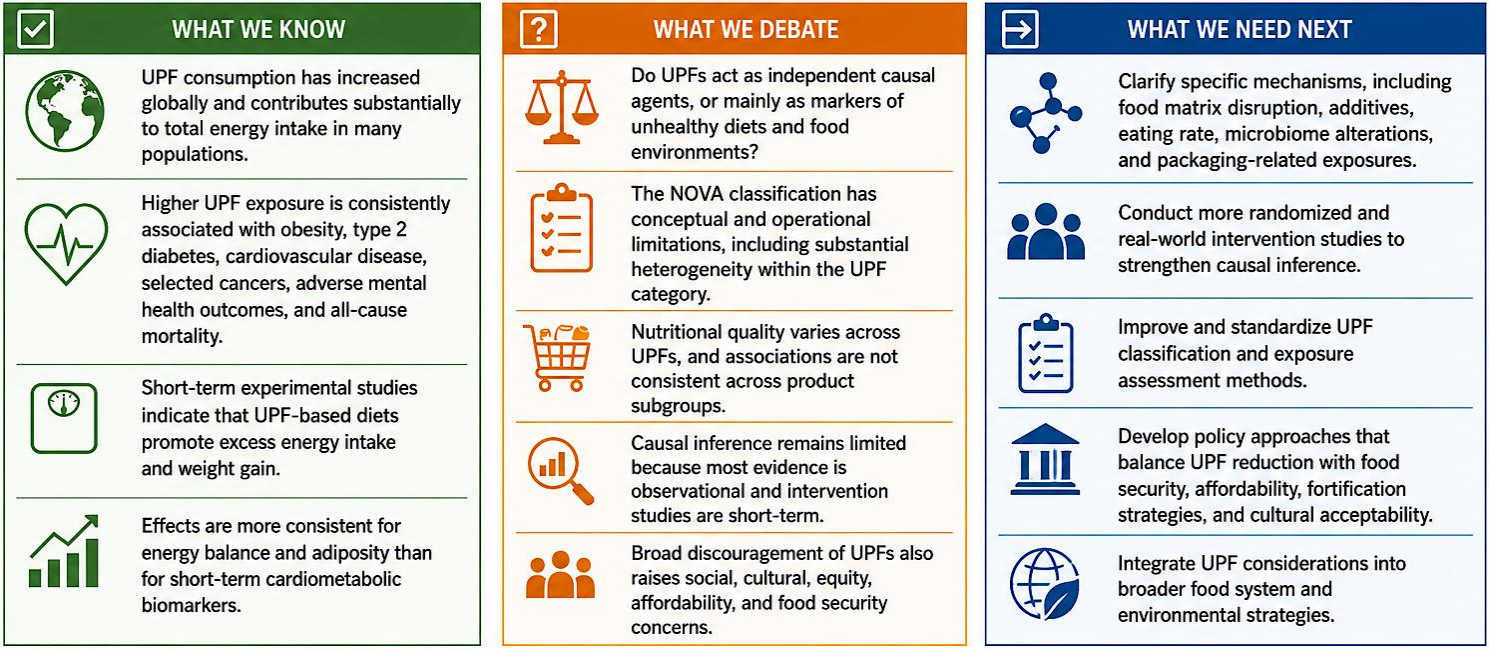
Summary of current evidence, ongoing debates, and future research priorities regarding UPFs and health

The growing scientific interest in UPFs reflects broader concerns about the transformation of contemporary food systems and dietary patterns. A synthesis of the current evidence, key controversies, and future research priorities is provided in Figure 2(Fig. 2).

Overall, the evidence reviewed in this manuscript indicates that UPF exposure is consistently associated with adverse health outcomes, while also highlighting that UPFs do not constitute a homogeneous category. Marked heterogeneity exists among products classified as ultra-processed, with some clearly characterized by poor nutritional quality and unfavorable health profiles, and others occupying a more ambiguous position from both nutritional and technological perspectives. This heterogeneity underlies both the explanatory value and the ongoing controversy surrounding the UPF concept.

Rather than being interpreted solely as unhealthy foods, UPFs may be better understood as indicators of prevailing modes of food production and distribution. They reflect systems that prioritize convenience, shelf stability, and sensory optimization, sometimes at the expense of nutritional adequacy. Their potential health effects are likely multifactorial, arising from a combination of high energy density, altered food structure, exposure to additives and processing-related compounds, and the progressive displacement of minimally processed foods from habitual diets. Therefore, the challenge extends beyond reducing UPF exposure per se to creating food environments in which minimally processed foods are accessible, affordable, culturally valued, and compatible with contemporary lifestyles. From this perspective, UPFs offer a helpful lens through which to reconsider the relationships among health, technology, and the organization of food production and exposure.

## Declaration

### Author contributions

Conceptualization: M.D., D.M., W.M.; Methodology: M.D., S.L., F.A., D.J.Z., W.M.; Data curation: S.L., F.A., D.J.Z., B.C., S.G.; Writing - original draft preparation: M.D., S.L., F.A., D.J.Z., S.G., W.M.; Writing - review and editing: All authors; Visualization: S.L., B.C.; Supervision: M.D., D.M., W.M.; Project administration: M.D. All authors have read and agreed to the published version of the manuscript.

### Funding

This research did not receive any specific grant from funding agencies in the public, commercial, or non-for-profit sectors. S.G. is funded by NHMRC Synergy Grant SOLVE CHD (#GNT1182301).

### Acknowledgments

M.D. and D.M. acknowledge the PRIN 2022 program of the Italian Ministry of University and Research (MUR), funded by the European Union-NextGenerationEU (Grant n: 2022HW2S5T).

The authors thank Melissa M. Lane for her valuable comments and insights during the revision of the manuscript.

### Conflict of interest

The authors declare no conflicts of interest.

### Artificial Intelligence (AI) - assisted technology

During the preparation of this manuscript, the authors used ChatGPT-5 to assist in editing the text and improving its readability. The authors have reviewed and edited the output and take full responsibility for the content of this publication.

### Institutional review board statement

Not applicable.

### Informed consent statement

Not applicable.

### Data availability statement

Not applicable.

## Figures and Tables

**Table 1:**
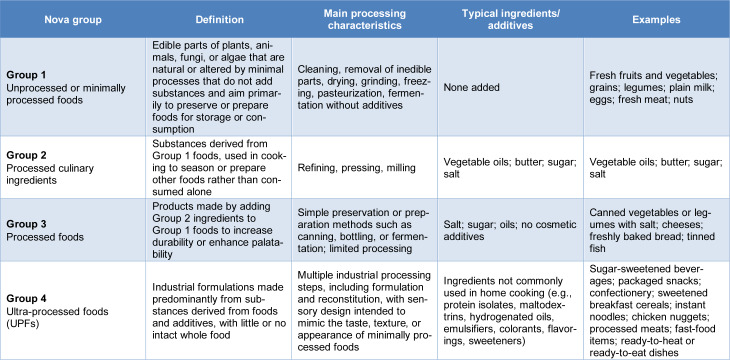
Nova classification system: definition and key characteristics

**Table 2:**
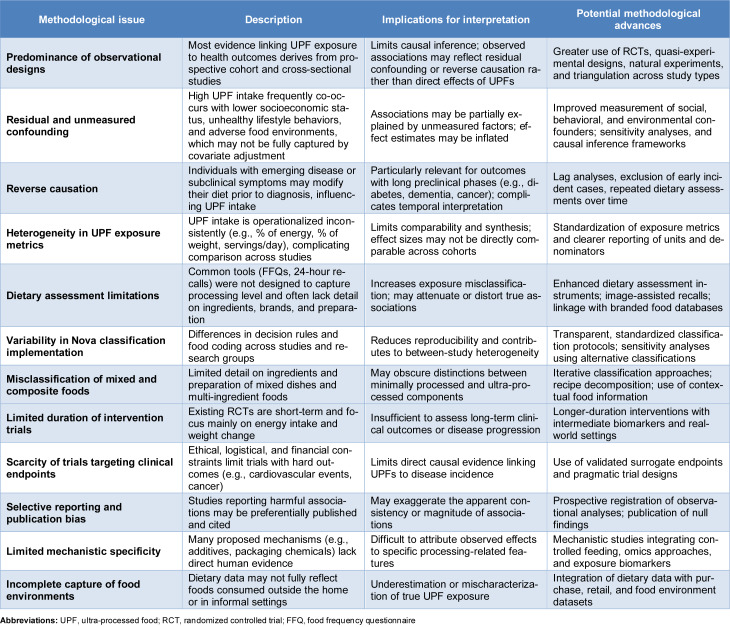
Key methodological limitations in UPF research, their implications, and potential methodological advances
